# Molecular characterization of endophytic and ectophytic plant growth promoting bacteria isolated from tomato plants (*Solanum lycopersicum* L.) grown in different soil types

**DOI:** 10.1186/s43141-022-00361-0

**Published:** 2022-05-24

**Authors:** Donia S. Helal, Hussein El-khawas, Tarek R. Elsayed

**Affiliations:** Department of Agricultural Microbiology, Faculty of Agriculture, Cairo University, Cairo, Egypt

**Keywords:** Tomato, PGPR, Endophytes, Soil type, Rhizocompetence, Genotypic diversity

## Abstract

**Background:**

Successful rhizosphere colonization by plant growth promoting rhizobacteria (PGPR) is of crucial importance to perform the desired plant growth promoting activities. Since rhizocompetence is a dynamic process influenced by surrounding environmental conditions. In the present study, we hypothesized that bacterial isolates obtained from different tomato plant microhabitats (balk soil, rhizosphere, endorhiza, phyllosphere, and endoshoot) grown in different soils (sand, clay, and peat moss) will show different rhizocompetence abilities.

**Results:**

To evaluate this hypothesis, bacterial isolates were obtained from different plant microhabitats and screened for their phosphate solubilizing and nitrogen fixing activates. BOX-PCR fingerprint profiles showed high genotypic diversity among the tested isolates and that same genotypes were shared between different soils and/or plant microhabitats. 16S rRNA gene sequences of 25 PGP isolates, representing different plant spheres and soil types, were affiliated to eight genera: *Enterobacter*, *Paraburkholderia*, *Klebsiella*, *Bacillus*, *Paenibacillus*, *Stenotrophomonas*, *Pseudomonas*, and *Kosakonia*. The rhizocompetence of each isolate was evaluated in the rhizosphere of tomato plants grown on a mixture of the three soils. Different genotypes of the same bacterial species displayed different rhizocompetence potentials. However, isolates obtained from the above-ground parts of the plant showed high rhizocompetence. In addition, biological control-related genes, *ituD* and *srfC*, were detected in the obtained spore forming bacterial isolates.

**Conclusion:**

This study evaluates, for the first time, the relationship between plant microhabitat and the rhizocompetence ability in tomato rhizosphere. The results indicated that soil type and plant sphere can influence both the genotypic diversity and rhizocompetence ability of the same bacterial species. Bacterial isolates obtained in this study are promising to be used as an environmentally friendly substitution of chemical fertilizers.

**Supplementary Information:**

The online version contains supplementary material available at 10.1186/s43141-022-00361-0.

## Background

Tomato (*Solanum lycopersicum)* is one of the most important vegetable crops in the world [1], where Egypt ranks in the fifth place regarding the world tomato production with 7 million tons/year (FAO, 2017). As the tomato plant is a highly demanding crop in terms of nutrients [2], a high amount of chemical fertilizers is normally applied to fulfill its nutrient requirements. However, it is well-known that the excessive application of chemical fertilizers increases its leaching and pollutes agricultural soil and water resources [3].

Plant growth-promoting rhizobacteria (PGPR) are widely applied as a substitute to chemical fertilizers to reduce negative impact on the agricultural ecosystem [4]. PGPR can promote plant growth via the production of essential substances [5], resulting from various processes such as fixation of atmospheric nitrogen [6], and the solubilization of phosphate, and potassium [7]. Moreover, PGPR can improve plant growth indirectly via the complex interaction between the host plant and its associated microbiota that can reduce the population of phytopathogens [8]. However, the ability of PGPR isolates to establish successful colonization in plant roots has been considered one of the most limiting factors that influence its effectiveness [9, 10].

Rhizocompetence is the ability of bacteria to colonize plant roots in natural soil and in the presence of endogenous soil microbiota. To establish a successful colonization, soil bacteria have to compete to benefit from the high-nutrient root exudates. Indeed, successful colonization with a certain population density is highly important to guarantee an effective plant growth-promoting activity [11]. Generally, rhizocompetence was correlated with traits such as siderophore production, substrate utilization, and denitrification [12]. Nevertheless, numerous factors can also influence the rhizocompetence ability and the survival rate of soil bacteria, such as active motility, biofilm formation, escape from predation, and adaptation to plant and soil edaphic factors [13, 14]. Furthermore, different bacterial genotypes can show different rhizocompetence abilities to specific plants [15] due to their specificity to plant root exudates [16, 17]. Additionally, some bacteria must first suppress plant immune responses to establish a successful colonization [18]. The soil type was thought to influence the rhizocompetence of introduced bacterial isolates, yet in the studies done by Schreiter et al. [19, 20]. They reported that the soil had a low or even no influence on the rhizosphere competence of *Pseudomonas* sp. RU47. On the other hand, Fließbach et al*.* [21] reported that *Pseudomonas fluorescens* colonization was more efficient in poorer microbial community soils than in richer soils. The application method can also affect the survival rate of inoculated isolates as reported by Götz et al. [22] where they stated that the root inoculation led to much better colonization by *Pseudomonas putida* and *Kosakonia cowanii* than seed inoculation.

Since rhizocompetence is influenced by these factors which can affect its survival rate after inoculation as biofertilizers, the major aim of this study is to characterize and evaluate the rhizocompetence ability of tomato associated bacteria with in vitro plant growth promoting activities. We hypothesized that bacterial isolates obtained from different tomato plant microhabitats and different soils will show different rhizocompetence ability when tested under greenhouse conditions. The prove of this hypothesis will improve our understanding of the fate of PGPR inoculums after field application.

## Methods

### Determination of plant growth-promoting population density in different plant spheres and soil types

#### Experimental design

Tomato seeds (*Solanum lycopersicum* cv. super strain-B) were cultivated by sowing in an experimental plot system with three different soil types in the experimental greenhouse of the Faculty of Agriculture, Cairo University, Giza, Egypt, for 2 months from June to September 2018. Each soil type was arranged in an independent experimental unit with three replicates, each pot of 15-cm diameter and 20-cm depth filled with about 1 kg of soil. The experiment was conducted at an open area (average temperature of 30–35 °C and 43–48% relative humidity). Pots were watered with the same volume of water every 2 days. A total of 9 pots were prepared, and each was eventually planted with 3 tomato seeds. The three soils (clay, sand, and peat moss) differed in their chemical and physical characteristics. The clay soil was obtained from a farm located at the Faculty of Agriculture, Cairo University (30° 01′ 09.5′′ N 31° 12′ 23.1′′ E), sand soil was obtained from a farm located at (30° 13′ 08.1′′ N 30° 29′ 34.6′′ E), and peat moss was obtained from SAB Syker Agarberatungs – und Handels GmbH& Co. (Plantaflor® SAB peat moss).

#### Sample preparation and determination of PGPR population densities

Tomato plants were harvested from each pot representing each soil type along with bulk soil samples at two time points (after 1 month, 1M; and after 2 months, 2M) and were processed to obtain different plant spheres (bulk soil, rhizosphere, endorhiza, endoshoot, and phyllosphere). Five grams of bulk soil from unplanted pots, the entire root after removing the loosely attached soil, and tomato phyllosphere were collected each in 45-ml saline solution (0.85% NaCl) and vigorously mixed at maximum speed using vortex for 1 min. The same root and phyllosphere samples were surface sterilized for 3 min in sodium hypochlorite solution (5% active chlorine) followed by 3% H_2_O_2_ for an additional 3 min, and finally, three washing steps for at least 10 min each using sterile 0.85% NaCl saline solution according to Sturz et al. [23]. Surface-sterilized plant samples were grinded in a sterile mortar and pestle. Tenfold serial dilutions of the obtained microbial suspensions, before and after surface sterilization, were prepared from each sphere, and 100 μl from each dilution was spread on the surface of each media (rich nutrient agar [24]; N-deficient combined carbon source medium (CCM), [25]); National Botanical Research Institute’s Phosphate (NBRIP) agar medium [26]) to obtain the population density of different PGPR fractions. Counts of colony-forming units (CFU) were estimated after 3 days of incubation at 30 °C for counting total bacteria and diazotrophs, then after 5 days of incubation for counting phosphate solubilizers. Bacterial colonies were considered as diazotrophs when grown on the nitrogen-free medium, while considered as phosphate solubilizers when surrounded by a clear zone after 5 days of incubation.

### Isolation of plant growth-promoting rhizobacteria (PGPR)

After estimating the PGPR population density in different plant spheres and soil types, the same plates were used for isolation purpose. Each plate, representing different media, was screened visually to isolate morphologically different bacterial colonies. The obtained isolates were purified by streaking several times on the same medium used for isolation. The ability to fix atmospheric nitrogen was confirmed after several steps of sub-culturing on CCM semi-solid nitrogen-free medium. Additionally, plant growth-promoting bacterial isolates were screened for their in vitro antibacterial and antifungal activity. The antimicrobial activity was evaluated according to Xue et al. [27] against *Ralstonia solanacearum* as a representative for bacterial phytopathogens. Nutrient agar medium (NA) was seeded, at 50 °C before solidification, with 10% of 24 h grown *R. solanacearum* culture then mixed and poured into Petri dishes. *R. solanacearum*-seeded medium was spot inoculated with each bacterial isolate and incubated at 30 °C for 24–48 h. Bacterial isolates surrounded by *R. solanacearum*-free zones were recorded as a positive result. The antifungal activities were tested against *Fusarium oxysporum* using dual culture plate assay. Potato dextrose agar medium (PDA) was inoculated by a 6-mm mycelial agar disc of 7-day-old fully grown *F. oxysporum* fungi at the center of the plate. A loopful of each of the bacterial isolates from an overnight culture was inoculated by streaking 3 cm away against the fungal mycelia disc. Plates were incubated for 5 days at 25 °C, and inhibited fungal growth was recorded as a positive result: either as a contact inhibition (C) or an inhibition zone (mm). Protease activity was estimated as well, by streaking bacterial isolates onto nutrient agar medium supplemented with skim milk (10%), the formation of clear zones considered as protease positive isolates. Bacterial isolates were preserved in Luria-Bertani broth (LB) [28] supplemented with 20% glycerol at −20 °C.

### Genomic DNA extraction from isolates

Bacterial isolates were grown in Luria-Bertani broth (LB) [28] for 24 h and were harvested by centrifugation at 12,000 g for 5 min after washing three times by resuspension in 0.85% NaCl and centrifugation. Genomic DNA was extracted using GeneJET Genomic DNA purification kit (Thermo Fisher Scientific, Lithuania) according to the manufacturer’s recommendations. DNA yields and purity were checked after agarose gel electrophoresis and ethidium bromide staining under UV light and NanoDrop spectrophotometer (NanoDrop 2000, Thermo Fisher Scientific, Germany). The DNA was stored at −20 °C.

### Genotypic diversity using BOX-PCR fingerprints

BOX-PCR fingerprints of bacterial isolates were generated for the strongest 77 plant growth-promoting rhizobacteria according to [29] using BOXA1R primer (Table 1). Eight microliters of the PCR products was separated by 1.5% agarose gel electrophoresis in 0.5 × TBE-buffer for 4 h (50 V). Gels were stained using ethidium bromide, then DNA was detected under UV light and BOX-PCR fingerprints patterns were analyzed and compared using the GelJ software v.2.0 [33]. The cluster analysis was performed using Pearson’s correlation coefficients and unweighted pair group method average (UPGMA) algorithm.

**Table 1:** Primers used in this study

Target gene	Primers used	sequence	size	Annealing temperature	Reference bacteria	Reference
**BOX A1**	**BOXA1 R**	CTACGGCAAGGCGACGCTGACG	Multiple sizes	53 °C	*P. fluorescens*	[29]
**16S rRNA**	**F-27** **R1494-1514**	AGA GTT TGA TC (AC) TGG CTC AG CTA CGG (T/C) TAC CTT GTT ACG AC	1400	56 °C	*P. fluorescens*	[30, 31]
**ItuD**	**ItuD1f** **ItuD1r**	GATGCGATCTCCTTGGATGT ATCGTCATGTGCTGCTTGAG	647	55 °C	*B. amyloliquefaciens*	[32]
**srfC**	**Sur3f** **Sur3r**	ACAGTATGGAGGCATGGTC TTCCGCCACTTTTTCAGTTT	441	55 °C	*B. amyloliquefaciens*	[32]

### Identification of PGP isolates using 16S rRNA gene sequencing

PCR amplification of the 16S rRNA gene fragments was performed using the bacterial-specific primers F-27 [30] and R1494-1514 [31] (Table 1) for 25 PGP bacteria representative for different spheres and sample types using thermal cycler PCR (Bio-Rad T100, USA). The PCR products were checked via agarose gel electrophoresis, then purified and sequenced by Macrogen (Seoul, Republic of Korea). Partial sequences of 16S rRNA genes were tested for similarity hits in the GenBank database using BLASTn (http://blast.ncbi.nlm.nih.gov/Blast.cgi). The 16S rRNA gene sequence of PGPR isolates was deposited in the NCBI GenBank database under the accession numbers (MT875283 to MT875304) and (MW269522 to MW269524).

### Phylogenetic analysis of PGPR isolates

The evolutionary history of the 25 PGPR bacterial isolates was inferred using the neighbor-joining method. The phylogenetic tree involved bacterial nucleotide sequences of which 25 sequences of 16S rRNA gene amplified from bacterial isolates of current study, while 46 sequences representing the closest hits were obtained from the NCBI GenBank database. The tree was computed using the maximum composite likelihood method, evolutionary analyses were conducted using MEGA version 5 software [34], and the phylogenetic tree architecture was confirmed via bootstrap analysis (1000 replicates) [35].

### Detection of plant growth-promoting and biological control-related genes for spore-forming isolates

Bacterial isolates were subjected to pasteurization step to select the spore forming bacteria that can tolerate the harsh environmental conditions in Egypt. PCR amplification of the *ituD* gene (encoding Iturin A) and *serC* gene (encoding surfactin) were performed for 11 spore-forming PGPR isolates using *Bacillus*-specific primers ItuD1f, ItuD1r for the amplification of *ituD* gene, and Sur3f, Sur3r for *srfC* gene (Table 1). The PCR products were checked via agarose gel electrophoresis.

### Genome mining analysis for the detection of antibiotic and secondary metabolite-related genes

Fourteen genome sequences representing the most similar hits of the identified bacterial isolates in this study were downloaded from the NCBI GenBank. Genome sequences were analyzed using the antiSMASH 5.0 (https://antismash.secondarymetabolites.org) an online platform [36] to detect genes encoding antibiotic and/or secondary metabolites in order to understand the potential mechanism which might be used by bacterial isolates for niche colonization and plant growth promotion.

### Rhizocompetence of in vitro PGPR isolates on tomato rhizosphere

The same tomato cultivar (*Lycopersicon esculentum*. cv. super strain-B) was used in this experiment to measure the ability of bacterial isolates to colonize tomato rhizosphere in the presence of indigenous soil microbiota. Twenty-one bacterial isolates, representing different plant spheres and soil types, were evaluated in a greenhouse experiment as follows:

#### Generation of antibiotic-resistant mutations

Initially, antibiotic-resistant mutations against rifampicin were induced for all tested isolates to facilitate their detection and enumeration by selective plating using a medium supplemented with this antibiotic. A volume of 100 μl of 24 h grown bacterial culture was plated onto nutrient agar medium supplemented with rifampicin (50 μg/ml). Rifampicin-resistant colonies (Rif^r^) were selected after 48 h, rechecked for their in vitro plant growth-promoting activities, and preserved at −20 °C in Luria-Bertani broth (LB) supplemented with 20% glycerol.

#### Greenhouse experiment

Bacterial cultures were prepared by inoculating 100-ml nutrient-broth medium supplemented with rifampicin (50 μg/ml). Bacterial cultures were centrifuged at 10,000×*g* for 5 min. After 72 h of incubation in a rotary shaker at 30 °C, the obtained cell pellets were washed three successive times using sterilized NaCl 0.85% solution, and the concentration of the bacterial cell cultures was adjusted to OD600 = 0.5 (about 10^6^ CFU/mL) using the same saline solution. Forty-day-old-tomato seedlings (*Solanum lycopersicum* cv. super strain-B) were soaked in the bacterial culture suspensions for 30 min. Inoculated tomato seedlings were transferred to pots filled with mixed soil (clay, sand, and peat moss, 1:1:1 v/v) in the experimental greenhouse of the Faculty of Agriculture, Cairo University, Giza, Egypt (16 h light and 28 °C). Each treatment was arranged in an independent experimental unit with three replicates, each pot of 15-cm diameter and 20-cm depth filled with about 1 kg of soil. Tomato plants were collected 30 days after inoculation (phenological stage R2 blister), where three plants per treatment were used to count the numbers of Rif^r^ inoculated bacteria. The entire root was transferred into Falcon tubes. CFU counts were enumerated by plating onto nutrient agar medium supplemented with rifampicin (50 μg/ml). Results were obtained after 48 h of incubation at 28 °C and related to gram root fresh mass (rfm).

### Data analysis

The greenhouse experiment rhizocompetence CFU counts mean and standard deviation were calculated using Microsoft Excel. The PCA analysis was performed using PAST4.03 software [37, 38].

## Results

### Effect of different plant spheres and soil types on plant growth-promoting population density

Viable counts of total bacteria, diazotrophs, and phosphate solubilizers were enumerated in different compartments of tomato plants grown in three different soil types after 1 and 2 months. The total viable counts of bacteria determined on nutrient agar medium of 1-month-old tomato plants showed different bacterial densities according to plant sphere. No significant differences (*P* < 0.05) were detected between the three bulk soil samples after 1 month, and the CFU counts ranged between (Log_10_ CFU g^-1^: 5.36 to 5.70), while significantly lower counts were detected for clay samples after 2 months compared to sand and peat moss (Log_10_ CFU g^-1^: 6.36, 7.16, and 7.55 for clay, sand, and peat moss, respectively). At the same time, the total population was significantly increased for all bulk soils at the second month (Table 2). A significantly higher bacterial population was detected in the rhizosphere of clay samples compared to those grown on sand or peat moss in both sampling times, whereas only clay rhizosphere samples were significantly increased after 2 months. Remarkably, higher bacterial populations were detected after 1 month in the endorhiza of peat moss samples compared to sand and clay (Log_10_ CFU g^-1^ = 7.06, 5.72, and 3.50, respectively), while at the last sampling time sand samples showed the highest endorhiza population (Log_10_ CFU g^-1^ = 6.15). No significant differences were detected among the bacterial population of tomato phyllosphere samples after 1 month, while significantly higher populations were detected in peat moss samples at the last sampling time. Notably higher diazotrophs populations were detected in the bulk soil of tomato plants grown on clay soil (Log10 CFU g^-1^ = 5.89) compared to those grown on sand or peat moss. However, on the rhizosphere samples, no outstanding differences were detected between clay and peat moss or between sand and peat moss. The only significant differences were noticed between clay and sand samples (Log10 CFU g^-1^ = 6.27 and 5.59, respectively). In the root endophytic compartments, no significant differences were detected between clay and sand samples; surprisingly, no diazotrophs were detected in rhizosphere samples of tomato plants grown on peat moss soil after 1 month while the diazotroph population reached 3.35 Log10 CFU g^-1^ root at the second sampling time. No significant differences in diazotroph population were detected between clay and sand or between sand and peat moss in phyllosphere. The only significant differences were detected between peat moss and clay (Log10 CFU g^-1^ = 7.38 and 6.69, respectively). Phyllosphere samples were characterized by the highest total bacterial and diazotrophs population among all tested spheres of tomato. Remarkably, higher phosphate solubilizing bacteria were detected in both bulk soil and rhizosphere samples of tomato plants growing on clay soil (Log10 CFU g^-1^ = 5.61 and 6.59, respectively) compared to sand and peat moss. No phosphate solubilizing bacteria were detected in root endophytic compartments neither in the phyllosphere, except for the phyllosphere of tomato plants grown on peat moss samples (Log10 CFU g^-1^ = 5.43). However, at the second sampling time, phosphate solubilizing bacteria were detected in phyllosphere (Table 2).

**Table 2:** Average CFU g^-1^ Log_10_ for total CFUs obtained from each plant sphere and soil type

Time	Sphere	TC			CCM			Phosphate solubilizers		
		Clay	Sand	Peat	Clay	Sand	Peat	Clay	Sand	Peat
**1 M**	**Bulk soil**	5.36^a^	5.70^a^	5.51^a^	5.89^a*^	5.22^b^	5.00^b^	5.61^a*^	4.74^b^	5.10^b^
	**Rhizosphere**	7.09 ± 0.18^a^	5.92 ± 0.28^b*^	6.34 ±0.56^b*^	6.27±0.1^a^	5.59 ± 0.36^b^	6.09±0.33^ab^	6.59±0.11^a*^	5.52±0.4^b*^	5.88±0.2^b*^
	**Endorhiza**	3.5± 0^a^	5.72 ± 2.15^b^	7.06± 0 ^c*^	3.5±0^a^	3.66±0.03^a*^	0±0^b^	0±0^a^	0±0^a^	0 ± 0^a^
	**Phyllosphere**	7.64 ± 0.11^a*^	7.67 ± 0.61^a*^	7.67 ± 0.57^a*^	6.69±0.43^b*^	7.02±0.34^ab*^	7.38±0.99^a*^	0±0^b^	0±0^b^	5.43±0^a^
**2 M**	**Bulk soil**	6.36 ^b*^	7.16^a*^	7.55^a*^	4.36^b^	5.24^a^	5.38^a^	3.43^b^	4.60^a^	4.93^a^
	**Rhizosphere**	8.46 ± 0.2^a*^	5.3 ± 0.02^b^	5.2 ± 0.35^b^	6.08±1.51^a^	5.74±0.31^a^	5.6±1.35^a^	4.75±0.58^a^	3.06±0^b^	4.23±0^c^
	**Endorhiza**	4.11 ± 0.06^b*^	6.15 ± 0.39^a^	5.28 ± 1.82^c^	4±0.51^a^	2.74±0^c^	3.35±0.17^b*^	0±0^a^	3.06±0^a^	0±0^a^
	**Phyllosphere**	5.97 ± 0.43^b^	5.72 ± 0.5^b^	7.1 ± 0.03^a^	5.18±0.72^b^	5.13±0.29^b^	5.8±0.18^a^	5.19±1.34^b*^	4.35±0.54^c*^	5.76±1.16^a^
	**Endoshoot**	6.32 ± 0.92^a^	4.94 ± 0.61^b^	6.35 ± 0.59^a^	3.75±0^b^	4.67±1.01^a^	4.99±0.19^a^	0±0^c^	2.81±0^b^	4.89±0.17^a^

Values marked with different letters are significantly different at (*p*< 0.05). Asterisks indicate significant differences between samples at different sampling times (1 month, 1M and 2 months, 2M)

### Isolation and characterization of bacteria with in vitro PGPR activity

A total of 489 bacterial isolates obtained from different tomato plant spheres and soil types (Table 3) were screened for their in vitro plant growth-promoting activities (nitrogen fixation, phosphate solubilization, and protease production and antimicrobial activities). The isolation procedure was accomplished based on selecting all morphologically different colonies from each petri dish, followed by preliminary screening of obtained isolates for their in vitro plant growth promoting activities.

**Table 3:** Total number of isolates obtained from different spheres and soil types using different isolation media

Soil type	Media\sphere	Bulk soil	Rhizosphere	Endorhiza	phyllosphere	Endoshoot	Total	%
**Sand**	**NA**	12	32	12	12	13	81	11.30%
	**CCM**	8	13	7	17	3	48	6.70%
	**Phosphate**	5	20	0	15	2	42	5.90%
**Clay**	**NA**	22	33	6	17	5	83	11.60%
	**CCM**	12	28	4	12	2	58	8.10%
	**Phosphate**	9	19	0	8	0	36	5.00%
**Peat**	**NA**	12	16	9	16	1	54	7.60%
	**CCM**	6	16	3	12	4	41	5.70%
	**Phosphate**	9	22	0	7	8	46	6.40%
**Total=489**		95	199	41	116	38	489	100%
		18.20%	40.80%	7.80%	26.60%	6.60%	100%	

A total of 124 bacterial isolates, obtained from the three soil types, showing notable phosphate solubilization activity was selected. The highest number of phosphate solubilizers was obtained from peat moss samples followed by sand (46 and 42 bacterial isolates, respectively), while only 36 phosphate solubilizing bacteria were isolated from clay samples (Table 3). Yet, within each soil type, different proportions of phosphate solubilizing bacteria were isolated from each plant sphere. Generally, the highest number of isolates was obtained from tomato plant ecto-spheres (rhizosphere and phyllosphere) followed by bulk soil. No phosphate solubilizing bacteria could be isolated from tomato endorhiza, while only two and eight bacterial isolates were isolated from the endoshoot of tomato plants grown on sand and peat moss soil, respectively.

A total of 147 potential diazotrophs isolates, able to grow on the nitrogen-free medium CCM, were selected. The highest number of diazotrophs was obtained from clay samples followed by sand (58 and 48 isolates, respectively), while only 41 bacterial isolates were isolated from peat moss samples. Again, the highest number of isolates was obtained from the rhizosphere and phyllosphere samples. Furthermore, a total of 218 bacterial isolates were selected from colonies grown on nutrient agar medium (Table 3). After isolation and visual evaluation of selected bacteria, the most promising isolates were selected based on the clear zone developed on phosphate solubilizing medium, or on the ability to grow after several sub-culture steps on N_2_-free semi-solid CCM medium. Finally, 77 bacterial isolates, representing different plant spheres and soil types, were selected for further evaluation (Table 4). Among those, 49 bacterial isolates representing (64%) were able to solubilize phosphate and grow on CCM medium, while only 6 and 9 isolates were either able to grow on CCM or solubilize phosphate, respectively. Moreover, bacterial isolates were screened for antagonistic activity against the phytopathogenic bacteria (*Ralstonia solanacearum*) and fungi (*Fusarium oxysporum*). Twenty-five bacterial isolates showed notable antifungal activity. Only 3 bacterial isolates showed antibacterial activity, while 7 showed antifungal and antibacterial activities as well. However, bacterial isolates showed different efficiencies in antagonistic actives (Table 4).

**Table 4:** Bacterial isolates and in vitro plant growth promoting related functions

Num	Key	NBRIP	CCM	AF (***F. oxysporum***) mm	AB (***R. solanacearum***) mm	Protease
1	TESHP-141	++	+	-	-	-
2	TESHP-144	+	-	-	-	-
3	TRP-11	+++	+	-	-	-
4	TRS-132	+	+	-	-	-
5	TESHP-142	+	+	-	-	++
6	TPHP-211	+	+	-	-	-
7	TPHP-213	+	+	-	-	-
8	TBP-50	++	+	-	-	-
9	TRP-45	+++	+	12	-	-
10	TRC-32	++	+	-	-	-
11	TESHP-143	++	+	C	1	-
12	TBP-49	++	+	-	2	-
13	TRP-13	+++	+	-	2	-
14	TRP-44	+++	+	16	-	+++
15	TRP-15	+++	+	10	1	-
16	TRS-75	+	+	-	-	-
17	TRP-49	+++	+	-	-	-
18	TRP-47	+++	+	8	-	+
19	TRP-46	+++	+	8	-	+
20	TBP-51	++	-	-	-	-
21	TPHS-100	++	-	15	-	+
22	TRC-57	+	+	-	-	-
23	TBC-8	+	+	-	-	-
24	TRS-133	+	+	-	-	-
25	TPHS-188	++	-	-	-	-
26	TRC-118	+	+	5	-	-
27	TRS-129	++	-	-	-	-
28	TBC-10P	+	+	-	-	+++
29	TPHS-203	+	+	3	2	+++
30	TRC-68	-	+	3	-	+
31	TBC-10	+	+	-	-	-
32	TBC-11	+	+	-	-	-
33	TRC-25	++	+	6	-	+
34	TRP-23	++	+	-	-	-
35	TRC-24	++	+	C	-	-
36	TRC-21	++	+	-	-	-
37	TBC-9	+	+	-	-	-
38	TRC-111	-	+	-	-	-
39	TESHP-145	+	+	-	-	-
40	TESHP-138	++	+	6	-	-
41	TRP-53	++	+	-	-	-
42	TRP-52	++	+	-	-	-
43	TRP-51	-	+	5	-	++
44	TRP-55	-	+	-	-	-
45	TRP-22	++	+	-	-	-
46	TERP-170	++	+	C	-	-
47	TRC-58	-	+	-	-	-
48	TERS-18	-	-	8	-	+
49	TERS-24	-	+	4	-	+
50	TERP-29	+	+	-	-	-
51	TERS-164	+	+	-	3	+++
52	TESHC-107	++	+	-	-	+++
53	TESHS-121	++	+	2	-	+
54	TPHS-84	+	-	-	-	-
55	TRP-26	++	+	-	-	-
56	TBP-41	+	+	C	-	-
57	TPHP-139	-	-	-	-	+++
58	TRP-31	+++	+	-	-	-
59	TPHS-86	-	-	-	-	+
60	TBP-42	++	-	-	-	-
61	TBP-43	-	-	-	-	-
62	TRS-155	+++	+	-	-	-
63	TRS-154	++	+	-	-	-
64	TPHS-205	+++	+	8	-	+
65	TBS-57	-	-	3	-	+
66	TPHP-137	+	+	-	-	-
67	TRC-5S	-	-	6	-	+
68	TRC-20S	+	-	5	-	+
69	TRC-8S	-	-	6	-	+
70	TRC-13S	-	-	9	-	+
71	TRC-23S	-	-	C	-	+++
72	TRC-22S	+	+	10	5	++
73	TRC-6S	++	-	13	-	++
74	TRC-27S	-	-	C	-	-
75	TRC-14S	-	-	15	3	+++
76	TRC-28S	-	-	C	4	++
77	TRC-29S	-	-	C	4	++

Key for letters: *T* tomato, *S* sand, *C* clay, *P* peat moss, *R* rhizosphere, *B* bulk soil, *PH* phyllosphere, *ER* endorhiza, *ESH* endoshoot; inhibition zone (C, inhibition at contact; mm) or (-) no inhibition zone, (-) no growth in CCM & NBRIP, (+) growth in CCM & growth without clear zone in NBRIP, (++) clear zone (1 to 5 mm), (+++) clear zone (5 to 10 mm) in NBRIP and protease test

### Genotypic diversity of plant growth promotion bacteria

**Fig. 1:**
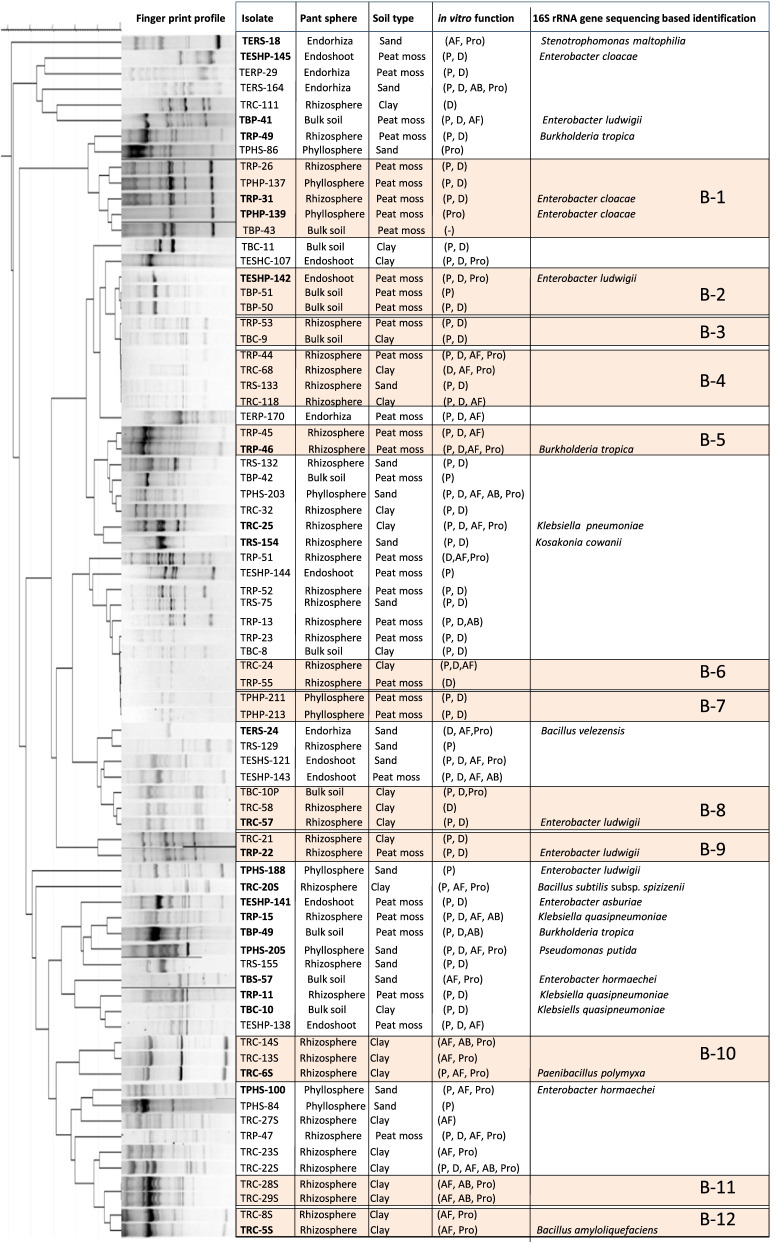
Dendrogram showing the UOGMA analysis between BOX PCR fingerprint profiles

The genotypic diversity of plant growth-promoting isolates was evaluated to determine the genotypes that are associated with different soils and plant spheres and that dominate different ecological niches. The evaluation also aimed to investigate whether isolates that share the same fingerprint profiles also have the same potentials and plant growth promotion-related functions. The BOX-PCR fingerprints of 77 PGPR isolates, which were selected after a second screening for in vitro PGPR activity, revealed a high genetic diversity among the tested isolates (Fig. 1). The fingerprint profiles allowed us to detect the presence of the same fingerprint profiles among different soils and/or plants spheres. About half of the tested bacterial isolates (32 isolates) formed 12 clusters with two or more BOX-PCR fingerprint profiles. The rest of the isolates (45 isolates) were unique genotypes each with only one fingerprint profile. Within each of the remaining clusters, identical fingerprint profiles were grouped together. Some of which were isolated from the same soil type and plant sphere, such as cluster 5 (TRP-45 and TRP-46) and cluster 7 (TPHP-211 and TPHP-213) of rhizosphere and phyllosphere samples of tomato plants grown on peat moss soil. Clusters 10, 11, and 12 all were isolated from the rhizosphere of tomato plants growing on clay soil. Other clusters represent bacterial isolates that were more specific to a particular soil but different plant spheres such as cluster 1 (TRP-26, TPHP-137, TRP-31, TPHP-139, and TBP-43) and cluster 2 (TESHP-142, TBP-51, and TBP-50), which were isolated from different spheres of tomato plants growing on peat moss soil. Cluster 8 (TBC-10p, TRC-58, and TRC-57) represents samples obtained from the clay soil. Bacterial isolates that were more specific to particular plant sphere irrespective of soil type such as cluster 4 (TRP-44, TRC-68, TRS-133, and TRC-118), cluster 6 (TRC-24 and TRP-55), and cluster 9 (TRC-21 and TRP-22) were all isolated from the rhizosphere samples of different soil types. Only cluster 3 (TRP-53 and TBC-9) represents different plant spheres, and different soil types in addition to the genotypes that were represented by only one individual (Fig. 1).

### Identification of bacterial isolates using 16S rRNA gene sequencing

**Fig. 2:**
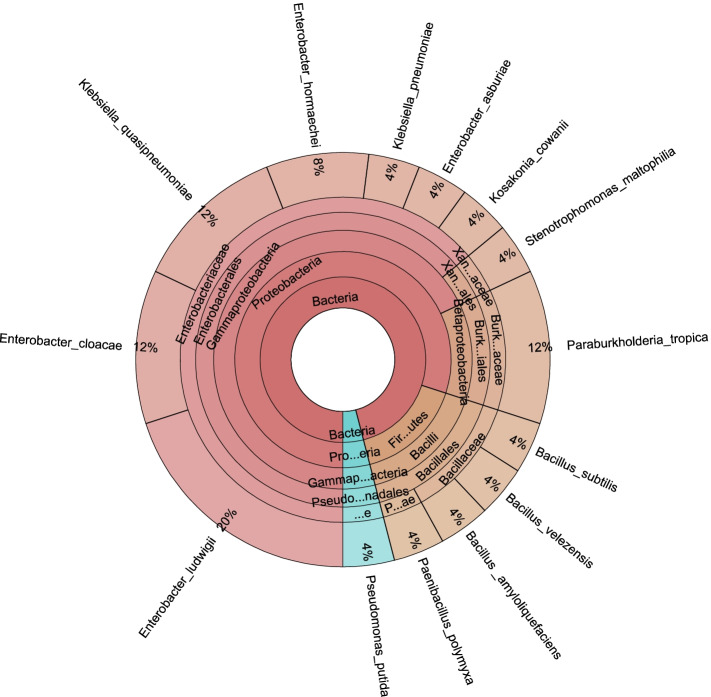
Krona graph showing the taxonomic affiliation of PGPR isolates

The 16S rRNA gene sequence analysis was performed on 25 bacterial isolates, which represent different BOX-PCR fingerprint profiles, to identify the most promising plant growth-promoting bacteria. The 16S rRNA gene sequence of 16 out of 25 (64%) was affiliated to the Enterobacteriaceae family. Eleven out of them were affiliated to the *Enterobacter* species (Fig. 2). Three isolates (TESHP-145, TPHP-139, and TRP-31) were 100% similar to *E. cloacae*, whereas five isolates (TPHS-188, TRC-57, TRP-22, TBP-41, and TESHP-142) were identified as *E. ludwigii* with 100% similarity except TESHP-142 which was 99.7%. Two isolates (TPHS-100 and TBS-57) were >99% similar to *E. hormaeche*, and only one was identified as *E. asburiae* (TESHP-141) with 99.9% similarity. Four isolates obtained from the bulk soil and tomato rhizosphere of clay and peat moss samples were identified with 100% similarity to the human pathogen *Klebsiella quasipneumoniae* and *Klebsiella pneumoniae* (TRP-11, TRP-15, TBC-10, and TRC-25). Only one isolate was identified as *Kosakonia cowanii* (TRS-154) with 99.9% similarity.

**Fig. 3:**
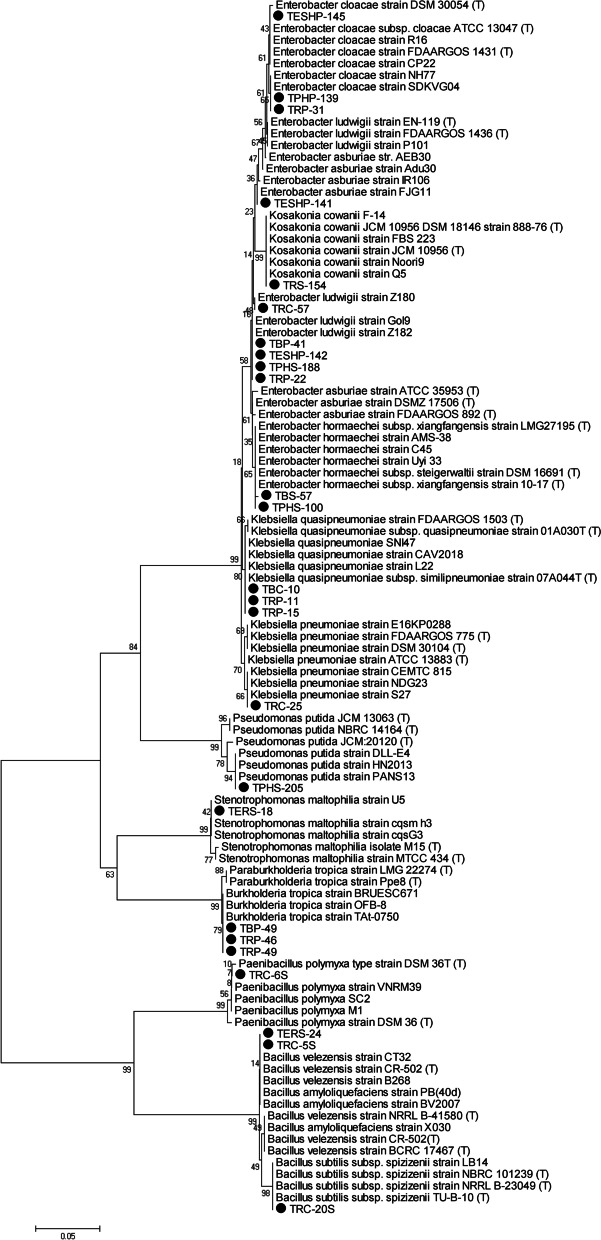
A neighbor-joining phylogenetic tree based on 16S-rRNA gene sequences. Dark circles represent bacterial isolates obtained in this study, and bootstrap values are indicated at each node

Furthermore, three out of 25 (12%) (TRP-46, TRP-49, and TBP-49) were affiliated to the Burkholderaceae family and obtained from the rhizosphere and bulk soil of peat moss samples. Those were identified as *B. tropica*, and the homotypic synonym is *Paraburkholderia tropica* with 100% similarity. Four isolates were Gram-positive spore-forming bacteria including three affiliated to the family Bacillaceae, *B. amyloliquefaciens* (TRC-5S), *B. subtillis* subsp. *spizizenii* (TRC-20S), *B. velezensis* (TERS-24), and one which was affiliated to the family Paenibacillaceae (*Paenibacillus polymyxa* TRC-6S). One bacterial isolate was identified as *Stenotrophomonas maltophilia* (TERS-18) with 99.9% similarity, and another was identified as *Pseudomonas putida* (TPHS-205) with 99.6% similarity (Table 5). The phylogenetic relatedness was confirmed in the neighbor joining tree (Fig. 3).

**Table 5:** Sequence analysis of 16S rRNA gene of PGPR isolated from different tomato plant spheres and soil types

Isolates	Identifications	Similarity %	Accession number
**TRC-5S**	*Bacillus amyloliquefaciens* strain PB (40d)	99.88	MT875302
**TRC-20S**	*Bacillus subtilis* subsp*. spizizenii* TU-B-10	100	MT875303
**TERS-24**	*Bacillus velezensis* strain B268	100	MT875295
**TRC-6S**	*Paenibacillus polymyxa* strain VNRM39	99.76	MT875304
**TBP-49**	*Burkholderia tropica* strain TAt-0750	100	MT875285
**TRP-49**	*Burkholderia tropica* strain TAt-0750	100	MT875287
**TRP-46**	*Burkholderia tropica* strain TAt-0750	100	MT875288
**TESHP-141**	*Enterobacter asburiae* strain IR106	99.87	MT875283
**TRP-31**	*Enterobacter cloacae* strain NH77	100	MT875298
**TESHP-145**	*Enterobacter cloacae* strain NH77	100	MT875293
**TPHP-139**	*Enterobacter cloacae* strain SDKVG04	100	MT875297
**TPHS-100**	*Enterobacter hormaechei* strain C45	99.78	MT875289
**TBS-57**	*Enterobacter hormaechei* strain C45	99.88	MT875301
**TPHS-188**	*Enterobacter ludwigii* strain Z182	100	MT875290
**TBP-41**	*Enterobacter ludwigii* strain Z182	100	MT875296
**TESHP-142**	*Enterobacter ludwigii* strain Z182	99.74	MW269522
**TRC-57**	*Enterobacter ludwigii* strain Z180	100	MW269524
**TRP-22**	*Enterobacter ludwigii* strain Z182	100	MW269523
**TRC-25**	*Klebsiella pneumoniae* strain CEMTC815	100	MT875292
**TRP-11**	*Klebsiella quasipneumoniae* strain CAV2018	100	MT875284
**TRP-15**	*Klebsiella quasipneumoniae* strain CAV2018	100	MT875286
**TBC-10**	*Klebsiella quasipneumoniae* strain CAV2018	100	MT875291
**TRS-154**	*Kosakonia cowanii* strain Noori9	99.85	MT875299
**TPHS-205**	*Pseudomonas putida* strain DLL-E4	99.63	MT875300
**TERS-18**	*Stenotrophomonas maltophilia* strain U5	99.89	MT875294

Isolate code letters: *T* Tomato, *S* Sand, *C* Clay, *P* Peat moss, *R* Rhizosphere, *B* Bulk soil, *PH* Phyllosphere, *ER* Endorhiza, *ESH* Endoshoot

### Genome mining of the closest genomes obtained from the NCBI GenBank for antibiotic and secondary metabolites related genes

Based on the BLAST results obtained from the NCBI GenBank, we performed a genome mining analysis using antiSMASH online platform, for the genomes closely related to our isolates, to identify potential antibiotics and secondary metabolite-related genes (Table S1). The analysis of the *Enterobacter* closest genome sequence (*E*. *cloacae* NH77, *E. ludwigii* P101, *E. asburiae* AEB30, and *E. hormaechei* C45) revealed the presence of genes encoding aryl polyene, colanic acid, and aerobactin. In addition, amonabactin was detected in the genome sequence of *E. ludwigii*. The analysis of *Kosakonia cowanii* strain FBS 223 revealed the presence of genes encoding colanic acid, carotenoid, and lipopolysaccharide. However, the comparison between *Klebsiella quasipneumoniae* strain CAV2018 and *Klebsiella pneumonia* strain E16KP0288 revealed the presence of genes encoding capsular polysaccharide and aerobactin, while only aryl polyene was detected on *K. quasipneumoniae.*

Nevertheless, the genome mining of *Stenotrophomonas maltophilia* strain U5 and *Paraburkholderia tropica* strain IAC135 showed the presence of genes encoding aryl polyene. Furthermore, the genome mining of *Bacillus* sp (*B. amyloliquefaciens* X030, *B. subtilis subsp. spizizenii* TU-B-10, and *B. velezensis* B268) revealed the presence of genes encoding the production of surfactin, bacillaene, bacillibactin, bacilysin, and teichuronic acid. In addition, fengycin, macrolactin H, and difficidin were present in both *B. amyloliquefaciens* and *B. velezensis*, while only *B. velezensis* showed in addition to the presence of gene encoding mersacidin. Meanwhile, the genome mining of *Paenibacillus polymyxa* SC2 revealed the presence of genes encoding (fusaricidin B, paenilan, and tridecaptin). Pseudopyronine was detected in the genome of *Pseudomonas putida* strain DLL-E4 (Table S1).

### Screening Bacillus isolates for genes potentially involved in plant microbe interaction

Gram positive spore-forming bacterial isolates were further investigated, by PCR for the presence of genes encoding antibacterial and antifungal compounds, such as Iturin A (*ituD*) and surfactin (*srfC*). Eight isolates showed positive results for *ituD* gene, five isolates were positive to *srfC*. *B. velezensis* (TERS-24), *B. amyloliquefaciens* (TRC-5S and TRC-8S), and *Paenibacillus polymyxa* (TRC-6s) were positive for the two tested genes (Table 6).

**Table 6:** *Bacillus* functional genes detected using PCR amplification

Bacterial isolate	Identification	***ituD***	***srfC***	Inhibition zone (mm) (***Fusarium oxysporum***)
**TERS-24**	*Bacillus velezensis*	+	+	4
**TRC-22S**	-	+	+	10
**TRC-5S**	*Bacillus amyloliquefaciens*	+	+	6
**TRC-6S**	*Paenibacillus polymyxa*	+	+	13
**TRC-8S**	*Bacillus amyloliquefaciens* (BOX-genotype)	+	+	6
**TRC-27S**	-	+	-	contact
**TRC-14S**	*Paenibacillus polymyxa* (BOX-genotype)	+	-	15
**TRC-13S**	*Paenibacillus polymyxa* (BOX-genotype)	-	-	9
**TRC-23S**	-	-	-	contact
**TRC-20S**	*Bacillus subtilis* subsp*. spizizenii*	+	-	5
**TRC-28S**	-	-	-	contact

### Evaluating the rhizocompetence of bacterial isolates in tomato rhizosphere

**Fig. 4:**
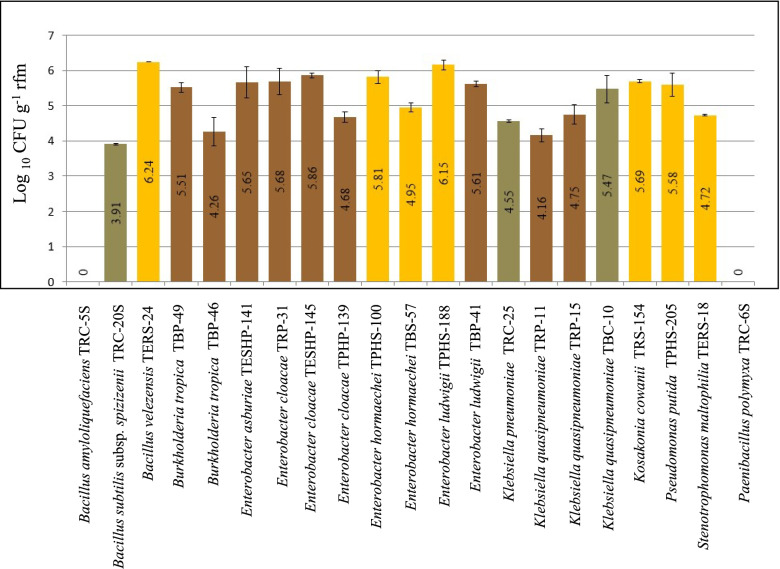
CFU counts of rifampicin-resistant PGPR isolates colonizing tomato root 1 month after inoculation

Rifampicin-resistant mutations (Rif^r^) were generated to facilitate their further detection in the rhizosphere samples. The rhizocompetence potentiality of 21 plant growth-promoting rhizobacteria on tomato rhizosphere was determined by CFU counts. The Rif^r^ CFU counts of PGPR 1 month after inoculation showed colonization densities ranging from no colonization to 6.24 (Log_10_ CFU g^-1^ rfm). Two PGPR isolates (TERS-24 and TPHS-188 isolated from the endorhiza and phyllosphere, respectively) were able to colonize tomato roots with a population density greater than 6 (Log_10_ CFU g^-1^ rfm). The population densities of 9 isolates were greater than 5 (Log_10_ CFU g^-1^ rfm) such as (TESHP-145, TPHS-100, TRS-154, TRP-31, TESHP-141, TBP-41, TPHS-205, TBP-49, and TBC-10) (Fig. 4).

## Discussion

### Effect of different plant spheres and soil types on PGPR population densities

The initial CFU counts of total bacteria, phosphate solubilizers, and diazotrophs showed that tomato plants grown in different soil types harbored different population densities, with higher numbers in rhizosphere samples compared to bulk soils, particularly in the rhizosphere of tomato plants grown in clay soil. Although no significant differences were detected between the three bulk soils regarding the total CFU counts, the clay soil was higher in phosphate solubilizers and diazotrophs. This result indicates that soil type has a great effect on the proportion of endogenous PGPR population density. However, in our previous study, Elsayed et al. [39], we found that the soil type slightly influenced the proportion and diversity of bacterial isolates with in vitro antagonistic activity towards *Ralstonia solanacearum*, while the plant sphere was the major driver. This could be attributed to the type of soils used in the present study, as we compared between three soil types, completely different in their chemical and physical structure, normally used during the commercial production of tomato (clay, sand, and peat moss). The clay soil is typically characterized by high organic carbon and nutrient content, which both have a large effect on the bacterial population density compared to sand [40–42]. The microbial density in rhizosphere is much higher compared to bulk soil [43], and this could explain the enrichment of PGPR in rhizosphere soil compared to its corresponding bulk soil. However, by analyzing tomato root endophytic compartments, we found that the higher bacterial population was detected in peat moss samples followed by sand, while clay samples were characterized by significantly lower bacterial populations. On the other hand, no significant differences were detected among the bacterial populations of phyllosphere samples. Generally, we can conclude that the effect of soil type mostly occurs in the spheres under the direct influence of soil such as the rhizosphere, while the soil effect is reduced in the above ground parts of the plant. This agrees with the previous study of Lundberg, [44] in which they reported that the microbial community in *Arabidopsis* plant compartments was strongly influenced by the soil type, while the endophytic compartments were characterized by overlapped low complexity microbial communities.

### Isolation and characterization of bacteria with in vitro PGPR activities

Over the course of this study, 489 bacterial isolates were obtained from different plant spheres and soil types. The genotypic diversity of 77 PGPR isolates, obtained from different plant spheres and soil types, was evaluated to determine the genotypes which dominate different ecological niches. Analysis of BOX-PCR fingerprint profiles revealed that within each cluster, identical fingerprint profiles were grouped together, whether they were isolated from the same soil type and/or plant sphere. Other clusters represent bacterial isolates that were more specific to a particular soil type irrespective of plant spheres (*E. cloacae* TRP-31, TPHP-139; cluster B-1). Some clusters represent bacterial isolates that were more specific to particular plant spheres irrespective of soil type (*E. ludwigii* TRP-22; cluster B-9). However, the preference of specific genotypes to a particular plant and/or soil was reported [41, 45].

### Identification of PGPR isolates using 16S rRNA gene sequencing

A total of 25 PGPR isolates were identified using 16S rRNA gene sequencing to study their phylogenetic relationship. The genus *Enterobacter* (represents 61% of the phylum Gammaproteobacteria obtained in the present study) was detected in almost all plant compartments and different soil types. It is frequently isolated from different spheres of diverse plants such as winter wheat phyllosphere [46], roots and leaves of banana [47], maize endophytic compartments [48], citrus plants [49], sweet potato [50], and soybean rhizosphere [51]. Furthermore, numerous reports have described the potentiality of *E. cloacae* to have plant growth-promoting activities [47, 52], which can enhance the growth of many plants such as soybean and wheat [51], due to its nitrogen fixation ability [53], antifungal suppression, phosphate solubilization, production of phytohormones, acetoin, and bioactive compounds [54]. *E. ludwigii* can fix atmospheric nitrogen and solubilize insoluble silicate and phosphate [55].

*E. hormaechei* has plant growth-promoting activities and was able to stimulate tomato root and shoot growth and alleviate salt stress, in addition to the production of different biological active compounds such as cell wall-degrading enzymes and IAA [56]. In this study, three isolates identified as *Klebsiella quasipneumoniae* and one as *Klebsiella pneumonia* were isolated from bulk soil and rhizosphere samples of tomato plants grown on peat moss and clay. *K. quasipneumoniae* can improve plant growth via the solubilization of inorganic phosphate [57]. However, it has the ability to adapt to both plant and clinical environments [58].

Only one isolate obtained in our study from the rhizosphere of tomato grown in sand soil was identified as *Kosakonia cowanii*. *Kosakonia* sp., which is an endophytic bacterium, able to fix atmospheric nitrogen with plant growth promoting activity on sugarcane and cereal crops [59]. One isolate was identified as *Stenotrophomonas maltophilia* obtained from root endophytic compartments of tomato plant grown on sandy soil. *S. maltophilia* is known for its ability to increase resistance against biotic and abiotic stress in wheat plants [60] and *Arachis hypogaea* [61]. On the other hand, *Pseudomonas putida* was obtained from tomato phyllosphere samples. It is known for its ability to stimulate plant growth via the production of growth regulators, antagonistic metabolites, phosphorus solubilization, and biological nitrogen fixation [62]. Three isolates were identified as *Burkholderia tropica* which is a nitrogen-fixing endophytic plant-associated bacterium commonly found in sugarcane [63].

The isolation of opportunistic human pathogens from plant rhizosphere was reported in numerous studies [64]. In our study, *Klebsiella pneumoniae* TRC-25 and *Klebsiella quasipneumoniae* TRP-11, TRP-15, and TBC-10 were isolated from bulk soil and rhizosphere samples. However, due to the notable overlap of traits identified as being important for colonization of the rhizosphere and animal tissues [65], using PGPR isolates with high similarity to opportunistic human pathogen should be avoided.

Only four isolates affiliated to the phylum Firmicutes were obtained (Bacillaceae, 3 isolates; and Paenibacillaceae, 1 isolate). *Paenibacillus polymyxa* was isolated from the rhizosphere of tomato plants grown on clay soil; it is an endophytic plant growth-promoting bacteria and efficient biocontrol agent against fungal wilt diseases [66]. *B. subtilis* can stimulate tomato seed germination and fruit quality via the production of auxins and its ability to solubilize insoluble phosphates [67]. *B. velezensis* and *B. amyloliquefaciens* can improve plant growth and induce resistance against phytopathogens [68].

### Screening Bacillus isolates for genes potentially involved in plant microbe interaction

The PCR amplification of antibiotic-related genes confirmed the presence of Iturin A (*ituD*), surfactin (*srfC*) in *B. amyloliquefaciens* (TRC-5S), and *B. velezensis* (TERS-24) as predicted from the genome mining analysis. Furthermore, the dual culture assay confirmed the in vitro antifungal activity against *F. oxysporum* (Table 6). Surfactin is known as a biocontrol agent, in addition to its vital role in motility, signaling, biofilm formation, and surface colonization [69]. Iturin is a lipopeptide antifungal compound that was identified as the most powerful fungicide [70]. Further genome mining analysis revealed the presence of fengycin, mycosubtilin, mersacidin, difficidin, bacillaene, and bacilysin which are considered antimicrobial metabolites [71–75]. Bacillibactin is a siderophore that contributes to the plant growth-promoting effects [76] while fengycin is associated with induced systemic resistance (ISR) [77]. The genome mining of *B. amyloliquefaciens* X030 and *B. velezensis* B268 revealed the presence of gene clusters coding for the biosynthesis of macrolactin, difficidin, and mersacidin all were reported to have an antimicrobial activity [74, 78]. Subtilosin A, subtilin, and mycosubtilin were only detected in *Bacillus subtilis* subsp. *Spizizenii* TU-B-10 genome, which has antimicrobial activities [75, 79]. This agrees with the study of Berendsen et al. [80] and supported by the fact that plants can recruit protective microorganisms and enhance microbial activity to suppress soil-borne pathogens.

### Evaluating the rhizocompetence of bacterial isolates in tomato rhizosphere

In this study, the highest rhizocompetence was detected for *Bacillus velezensis* TERS-24 (6.24 Log_10_ CFU g^-1^rfm). It was reported that cyclic lipopeptides such as surfactin produced by *Bacillus* spp. can trigger the formation of biofilm which is an essential step in root colonization [81]. However, while surfactin non-producing *B. velezensis* FZB42 mutant only showed a slight difference in the colonization, exopolysaccharide non-producing mutant had completely lost its ability to form biofilm and was unable to colonize tomato rhizosphere efficiently [82]. On the other hand, *Paenibacillus polymyxa* TRC-6S did not show any rhizocompetence and was under detection limit on tomato rhizosphere after 1 month, possibly owing to its colonization patterns as a leaf-inhabiting endophyte [83]. Nevertheless, the rhizocompetence of *B. velezensis* can be affected by the presence of other microorganisms in soil as reported by Abdallah et al. [84]. They found that the colonization level of *B. velezensis* was highly improved in tomato rhizosphere by the presence of *Agrobacterium tumefaciens*, and they attributed that to the modulations on tomato root exudates. *Bacillus subtilis* subsp. *spizizenii* TRC-20S obtained in this study showed a relatively lower population on tomato rhizosphere (3.9 Log_10_ CFU g^–1^ rfm), while *B. amyloliuefaciens* TRC-5S was below the detection limit. Furthermore, *Pseudomonas putida* TPHS-205 isolated from the phyllosphere of tomato plants grown on sand soil sowed high rhizocompetence (5.58 Log_10_ CFU g^–1^ rfm). This result agrees with our previous study Elsayed et al. [8] as we reported that *Bacillus velezensis* (B63) had much lower rhizocompetence ability compared to *Pseudomonas fluorescens* (P142) on tomato rhizosphere (3.1 and 5.9 Log_10_ CFU g^–1^ rfm, respectively). The rhizocompetence of *Burkholderia tropica* TBP-49 (Log_10_ CFU g^–1^ rfm = 5.51) surpassed that of *Burkholderia tropica* TRP-46 (Log_10_ CFU g^–1^ rfm = 4.26). Although both strains were isolated from peat moss samples and showed 100% 16S rRNA gene similarity to the nitrogen fixing *B. tropica* strain TAt-0750 recovered from a tomato plant [85], they showed different fingerprint profiles. In addition, *B. tropica* TBP-49 showed antibacterial activity while *B. tropica* TRP-46 showed only antifungal activity which could explain the proliferation of *B. tropica* TBP-49 in tomato rhizosphere. This is in agreement with the previous study of Ghirardi et al. [12], where they reported that the rhizocompetence was associated with the ability to produce antibiotics. The differences in plant growth promotion among the isolates are attributed to their individual competencies [86]. It is reported that PGPRs colonize more efficient in poorer microbial communities than in richer soils [21].

**Fig. 5:**
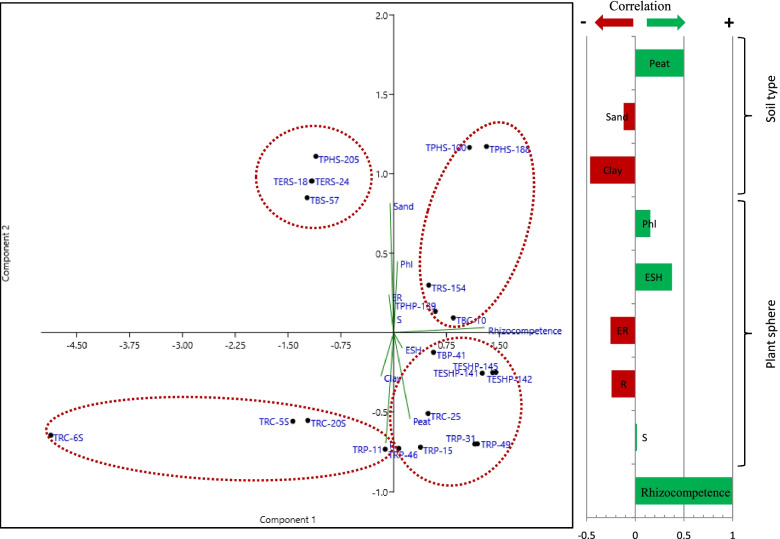
PCA analysis showing the correlation between rhizocompetence potential and the origin of bacterial isolates

The highest rhizocompetence recorded in our study was for *B. velezensis* TERS-24 and *E. ludwigii* TPHS-188, which were isolated from tomato plants grown on sandy soil. Surprisingly, the principal component analysis (PCA) (Fig. 5), showing the correlation between rhizocompetence potential and the origin of bacterial isolates, revealed a positive correlation between rhizocompetence and isolates obtained from the above-ground parts of plant. Nevertheless, further genome mining analysis of *E. cloacae* NH77, *E. ludwigii* P101, *E. asburiae* AEB30, and *E. hormaechei* C45, which has the highest similarity to *Enterobacter* isolates obtained in this study (Table S1), revealed the presence of gene clusters coding for the biosynthesis of aryl polyene, colanic acid, and aerobactin which can play a major role in plant root colonization. Aryl polyene is a polyunsaturated lipid that allows bacteria to form biofilm and adhere to surfaces [87]. It is also involved in colonization [88]. Colanic acid is an exopolysaccharide critical for biofilm formation and survival on plants [89]. Aerobactin is a siderophore with antagonistic activity against several soil-borne pathogens [90]. The genome mining analysis of both *K. quasipneumoniae* strain CAV2018 and *K. pneumonia* strain E16KP0288, revealed the presence of genes encoding capsular polysaccharide production, which allows the bacteria to survive under various environmental stresses [91]. However, aryl polyene was also detected only on *K. quasipneumoniae.* Furthermore, the genome mining of *Kosakonia cowanii* strain FBS 223 revealed genes encoding carotenoids and colonic acid. Carotenoids can support the survival of bacteria in rhizosphere [92] and can promote both plant growth and defense against pathogens [93]. To our knowledge, no studies have been conducted to investigate whether the source of isolate contributes to its rhizocompetence ability.

## Conclusion

This study showed that soil type and plant sphere can influence both population density and genotypic diversity of plant growth-promoting bacteria associated with tomato plants. However, a tissue- and soil-specific genotypes could be detected. Furthermore, different genotypes of the same bacterial species can have different rhizocompetence potentials. The PCR amplification of antibiotic-related genes confirmed the presence of Iturin A (*ituD*), surfactin (*srfC*) in Gram positive isolates as predicted from the genome mining analysis. All these factors should be taken into consideration when the isolates are used as bio-preparations such as biofertilizers or biological control products to assure the successful colonization of the host plant. Several isolates obtained in this study have great promises as plant growth promoting inoculants due to their in vitro activities as well as their high rhizocompetence abilities.

## Supplementary Information

**Additional file 1: Table S1.** Antibiotics and secondary metabolites related genes as detected in the genome sequences of closely related genomes to the isolates obtained from this study.

## Data Availability

The authors declare that all data supporting the findings of this study are included within the article.

## Abbreviations

PGPR: Plant growth promotion rhizobacteria
CCM: Composition of N-deficient combined carbon sources soli medium
NBRIP: National Botanical Research Institute’s Phosphate
CFU: Colony-forming unit
NA: Nutrient agar medium
PDA: Potato dextrose agar
LB: Laurai-Bretani broth
Rif^r^: Rifampicin-resistant colonies
OD600: Optical density measured at a wavelength of 600 nm
rfm: Root fresh mass
TC: Total count
AF: Antifungal activity
AB: Antibacterial activity
T: Tomato
S: Sand
C: Clay
P: Peat moss
R: Rhizosphere
B: Bulk soil
PH: Phyllosphere
ER: Endorhiza
ESH: Endoshoot
IAA: Indole-3-acetic acid
PCR: Polymerase chain reaction
ISR: Induced systemic resistance
PCA: Principal component analysis
C: Contact inhibition
mm: An inhibition zone
