# Supplementary material for: Molecular characterization of endophytic and ectophytic plant growth promoting bacteria isolated from tomato plants (Solanum lycopersicum L.) grown in different soil types
Source: J Genet Eng Biotechnol. 2022 May 24;20:79. doi: 10.1186/s43141-022-00361-0 (PMC9130443; doi:10.1186/s43141-022-00361-0)
Supplement: Supplementary file 1 — Additional file 1: Table S1. Antibiotics and secondary metabolites related genes as detected in the genome sequences of closely related genomes to the isolates obtained from this study. [file 43141_2022_361_MOESM1_ESM.docx]

| **Predicted metabolites** | *Bacillus amyloliquefaciens* strain X030  (99.76 % to TRC-5S) | *Bacillus subtilis* subsp*. spizizenii*  TU-B-10 (100% to TRC-20S) | *Bacillus velezensis* strain B268  (100% to TERS-24) | *Paenibacillus polymyxa* SC2  (99.64% to TRC-6S) | *Enterobacter cloacae* strain NH77  (100% to TESHP-145 & TRP-31,  and 99.88 % to TPHP-139) | *Enterobacter ludwigii* strain P101  (99.41 to TRC-57, 99.29 to TRP-22, 99.24% to  TPHS-188, 99.09 to TBP-41, and 98.8 to TESHP-142) | *Enterobacter asburiae* str. AEB30  (99.60 to TESHP-141) | *Enterobacter hormaechei* strain C45  (99.88 % to TBS-57, and 99.78 % to TPHS-100) | *Klebsiella quasipneumoniae* strain CAV2018  (100% to TBC 10, TRP-15 and, TRP-11) | *Klebsiella pneumonia* strain E16KP0288  (99.89 % to TRC-25) | *Kosakonia cowanii* strain FBS 223  (99.70 % to TRS-154) | *Stenotrophomonas maltophilia* strain U5  (99.89 % to TERS-18) | *Pseudomonas putida* strain DLL-E4  (99.63 % to TPHS-205) | *Paraburkholderia tropica* strain IAC135 -  (99.66% to TRB-49, 99.67 % to TBP-49  , and 99.48 % to TRP-46) |
| --- | --- | --- | --- | --- | --- | --- | --- | --- | --- | --- | --- | --- | --- | --- |
| **Aryl polyene** | - | - | - | - | 83% | 100% | 100% | 88% | 100% | - | - | 35% | - | 35% |
| **Colanic acid** | - | - | - | - | 100% | 100% | 100% | 100% | - | - | 100% | - | - | - |
| **Surfactin** | 82% | 73% | 82% | - | 8% | 8% | - | - | - | - | - | - | - | 8% |
| **Aerobactin** | - | - | - | - | 66% | 66% | 66% | 66% | 22% | 22% | - | - | - | - |
| **Capsular polysaccharide** | - | - | - | - | - | - | - | 20% | 53% | 60% | - | - | - | - |
| **Amonabactin P 750** | - | - | - | - | - | 57% | - | - | - | - | - | - | - | - |
| **Carotenoid** | - | - | - | - | - | - | - | - | - | - | 100% | - | - | - |
| **Lipopolysaccharide oligosaccharide** | - | - | - | - | - | - | - | - | - | - | 80% | - | - | - |
| **Fusaricidin B** | - | - | - | 100% | - | - | - | - | - | - | - | - | - | - |
| **Paenilan** | - | - | - | 100% | - | - | - | - | - | - | - | - | - | - |
| **Iturin** | 22% | - | 22% | - | - | - | - | - | - | - | - | - | - | - |
| **Tridecaptin** | - | - | - | 80% | - | - | - | - | - | - | - | - | - | - |
| **Bacillaene** | 100% | 100% | 100% | - | - | - | - | - | - | - | - | - | - | - |
| **Fengycin** | 100% | - | 100% | - | - | - | - | - | - | - | - | - | - | - |
| **Bacillibactin** | 100% | 100% | 100% | - | - | - | - | - | - | - | - | - | - | - |
| **Bacilysin** | 100% | 100% | 100% | - | - | - | - | - | - | - | - | - | - | - |
| **Teichuronic acid** | 100% | 100% | 100% | - | - | - | - | - | - | - | - | - | - | - |
| **Subtilosin A** | - | 100% | - | - | - | - | - | - | - | - | - | - | - | - |
| **Subtilin** | - | 92% | - | - | - | - | - | - | - | - | - | - | - | - |
| **Mycosubtilin** | - | 100% | - | - | - | - | - | - | - | - | - | - | - | - |
| **Macrolactin H** | 100% | - | 100% | - | - | - | - | - | - | - | - | - | - | - |
| **Difficidin** | 100% | - | 100% | - | - | - | - | - | - | - | - | - | - | - |
| **Mersacidin** |  | - | 100% | - | - | - | - | - | - | - | - | - | - | - |
| **Pseudopyronine** | - | - | - | - | 12% | - | - | - | - | - | - | - | 62% | 18% |

Table S1. Antibiotics and secondary metabolites related genes as detected in the genome

sequences of closely related genomes to the isolates obtained from this study
